# Dynamic changes in short- and long-term bacterial composition following fecal microbiota transplantation for recurrent *Clostridium difficile* infection

**DOI:** 10.1186/s40168-015-0070-0

**Published:** 2015-03-30

**Authors:** Alexa Weingarden, Antonio González, Yoshiki Vázquez-Baeza, Sophie Weiss, Gregory Humphry, Donna Berg-Lyons, Dan Knights, Tatsuya Unno, Aleh Bobr, Johnthomas Kang, Alexander Khoruts, Rob Knight, Michael J Sadowsky

**Affiliations:** Department of Soil, Water, and Climate, and Microbial and Plant Genomics Institute, University of Minnesota, St Paul, MN USA; BioFrontiers Institute, University of Colorado, Boulder, CO USA; Department of Computer Science, University of Colorado, Boulder, CO USA; Department of Chemical and Biological Engineering, University of Colorado, Boulder, CO USA; Cooperative Institute for Research in Environmental Sciences, University of Colorado, Boulder, USA; Department of Computer Science and Engineering, University of Minnesota, Minneapolis, MN USA; BioTechnology Institute, 1479 Gortner Ave, 140 Gortner Labs, St. Paul, MN 55108 USA; Division of Gastroenterology, Department of Medicine, Center for Immunology, University of Minnesota, Minneapolis, MN USA; Department of Chemistry & Biochemistry, University of Colorado, Boulder, CO USA; Howard Hughes Medical Institute, Boulder, CO USA

**Keywords:** Short- and long-term changes in microbiota following FMT

## Abstract

**Background:**

Fecal microbiota transplantation (FMT) is an effective treatment for recurrent *Clostridium difficile* infection (CDI) that often fails standard antibiotic therapy. Despite its widespread recent use, however, little is known about the stability of the fecal microbiota following FMT.

**Results:**

Here we report on short- and long-term changes and provide kinetic visualization of fecal microbiota composition in patients with multiply recurrent CDI that were refractory to antibiotic therapy and treated using FMT. Fecal samples were collected from four patients before and up to 151 days after FMT, with daily collections until 28 days and weekly collections until 84 days post-FMT. The composition of fecal bacteria was characterized using high throughput 16S rRNA gene sequence analysis, compared to microbiota across body sites in the Human Microbiome Project (HMP) database, and visualized in a movie-like, kinetic format. FMT resulted in rapid normalization of bacterial fecal sample composition from a markedly dysbiotic state to one representative of normal fecal microbiota. While the microbiome appeared most similar to the donor implant material 1 day post-FMT, the composition diverged variably at later time points. The donor microbiota composition also varied over time. However, both post-FMT and donor samples remained within the larger cloud of fecal microbiota characterized as healthy by the HMP.

**Conclusions:**

Dynamic behavior is an intrinsic property of normal fecal microbiota and should be accounted for in comparing microbial communities among normal individuals and those with disease states. This also suggests that more frequent sample analyses are needed in order to properly assess success of FMT procedures.

**Electronic supplementary material:**

The online version of this article (doi:10.1186/s40168-015-0070-0) contains supplementary material, which is available to authorized users.

## Background

Fecal microbiota transplantation (FMT) has emerged in recent years as a highly effective treatment for refractory *Clostridium difficile* infection (CDI) that cannot be cured with antibiotics alone [1]. The procedure leads to prompt engraftment of donor microbiota, attainment of donor-like bacterial diversity, and normalization of the overall microbial community structure [2-8]. However, existing data characterizing long-term stability of engrafted microbiota are limited. One recent study suggests the microbiota of patients after FMT may not fully recover until 16 weeks after the procedure [9]. This type of analysis, however, is complicated by the fact that the microbial communities are intrinsically dynamic and affected by daily fluctuations in the host’s diet, activities, and health [10-12]. In addition, multiple fixed host factors, such as different states of immune competence, genetics, or gastrointestinal anatomy, likely also affect the composition, stability, or resilience of colonic microbiota [13-17]. Therefore, it is unclear whether divergence in post-FMT microbiota from that of donor implant material represents continued recovery, or whether these temporal changes are a general characteristic of host-associated gut microbiota in a changing host environment.

Here we describe both short- and long-term dynamic changes of fecal bacterial composition in four patients following FMT. All patients received microbiota from the same pre-qualified donor according to the standardized FMT protocol we described previously [18]. Three patients received freshly prepared microbiota and one patient received microbiota that had previously been frozen. We compared pre- and post-FMT fecal microbial communities from these patients, as well as pre-FMT communities from 10 additional patients with multiply recurrent CDI (R-CDI), to the sequences of normal subjects described in the Human Microbiome Project [19]. In addition, we compared temporal changes in fecal bacterial composition in recipients following FMT to temporal changes observed within samples from the donor.

## Results

### Bacterial composition of fecal samples from patients with recurrent CDI becomes healthy and donor-like following FMT

Four patients (CD1 to CD4) with recurrent CDI were treated with FMT using material obtained from a single donor but from different time points, and fecal samples were collected from these patients before and after the procedure as well as from the donor at times of donation. Bacterial communities from these fecal samples were characterized by sequencing the V4 region of the 16S rRNA gene. Following trimming and quality filtering from a total of 12,536,492 sequences, we randomly subsampled to 5,000 sequences/sample in order to normalize read depth across all samples. All further analyses were performed using this rarefied read depth.

**Figure 1:**
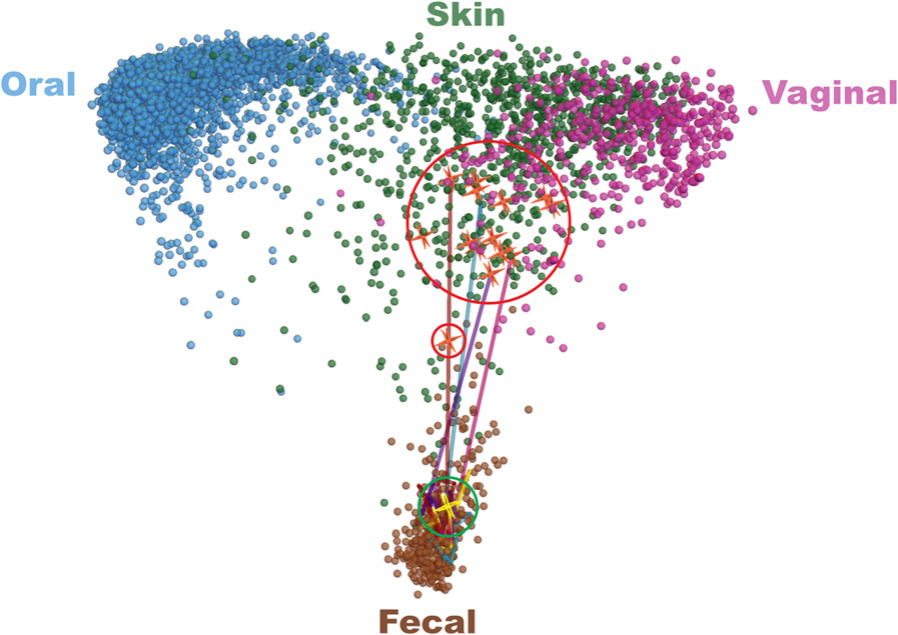
**Fecal bacterial communities of recurrent CDI patients shift towards HMP fecal bacterial communities after FMT.** Pre-FMT patient samples (red circle); post-FMT patient samples (green circles); trajectory of patient fecal communities after FMT (blue line).

To better understand changes in bacterial communities following FMT, we compared the bacterial composition of patient fecal samples to those of microbial communities from various body sites from the 252 healthy individuals characterized in the Human Microbiota Project (HMP) [19] (Figure 1) using unweighted UniFrac [20] followed by principal coordinates analysis (PCoA) [21] (see Additional file 1: Movie supplement). The composition of pre-FMT fecal samples from patients CD1 to CD4 and 10 additional patients with recurrent CDI was distinct from both fecal samples from healthy individuals and microbial communities at other body sites, including mouth, vagina, and skin, demonstrating severe alterations in pre-FMT communities compared to healthy fecal communities as has been previously shown [4,5]. In contrast, microbial communities from the donor fell within the range of healthy fecal samples. Using an animated visualization of FMT-associated changes in patients’ fecal microbial communities, we observed rapid and dramatic shifts after FMT towards the communities found in the feces of healthy individuals and of the original donor (see Additional file 1: Movie supplement).

### Fecal microbial communities remain dynamic following FMT

**Figure 2:**
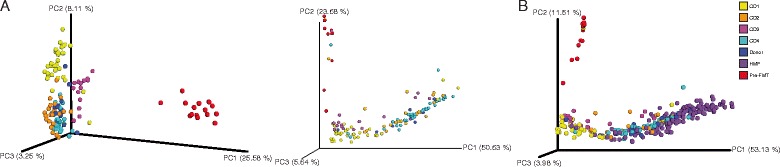
**Microbial communities shift following FMT. (A)** Unweighted (left) and weighted (right) UniFrac analyses followed by principal component analysis of bacterial communities of recurrent CDI patient fecal samples before (red) and after FMT and donor samples (blue). **(B)** Weighted UniFrac analysis followed by principal component analysis of bacterial communities of patients before (red) and after FMT versus HMP fecal communities (purple). PC, principal component. Percentages represent percent variability explained by each principal component. Se key at right for colors associated with samples before FMT (pre-FMT), from HMP and donor, and from patients after FMT (CD1 to CD4).

To more closely examine temporal changes in recipient fecal samples following FMT, we analyzed fecal microbial communities from patients CD1 to CD4 and donor, as well as from 10 additional donor samples, using weighted and unweighted UniFrac [20] followed by PCoA [21]. This analysis demonstrated that fecal bacterial communities continued to undergo compositional fluctuation following FMT (Figure 2A and Additional file 2: Figure S1; individuals OTUs listed in Additional file 3: Table S1).

To determine whether this dynamic range of post-FMT microbial composition fits within the range seen across healthy individuals, we also compared communities in our samples to those in the HMP *via* weighted UniFrac and PCoA (Figure 2B). Again, fecal microbial communities prior to FMT were highly distinct from healthy fecal microbial communities, and following the procedure, these communities more closely resembled those of healthy individuals. Similar to the comparison with donor communities above, fecal microbial communities of recurrent CDI patients following FMT shifted within the cluster of communities from healthy individuals.

### Rapid and substantial changes to *Enterobacteriales* in feces following FMT

**Figure 3:**
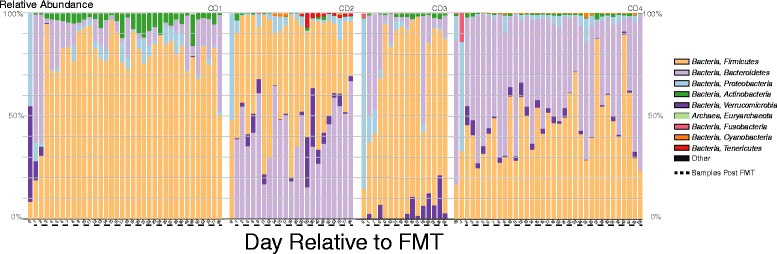
**Changes in fecal microbial communities after FMT.** Relative abundance of sequences classified to the level of bacterial phyla before and after FMT in patient fecal samples. Samples after FMT indicated with dashed line. See key at right.

**Figure 4:**
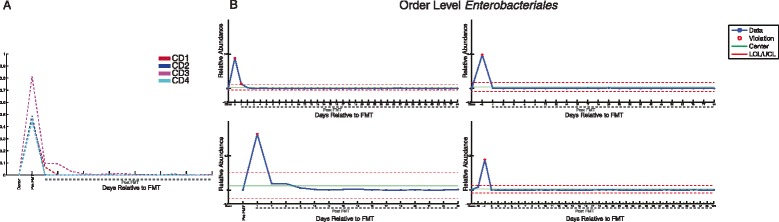
**Changes in the order** ***Enterobacteriales*** **after FMT. (A)** Relative abundance of *Enterobacteriales* in donor and patient samples before and after FMT in samples common across all patients. **(B)** Control charts of relative abundance of *Enterobacteriales* in donor (leftmost sample) and patient samples before and after FMT. Patient CD1 (top left), patient CD2 (top right), patient CD3 (bottom left), patient CD4 (bottom right). LCL, lower control limit; UCL, upper control limit; mean relative abundance in all samples (center). LCL and UCL represent three standard deviations in relative abundance below and above the mean, respectively. Dashed lines indicate samples after FMT.

While overall fecal microbial communities were dramatically altered following FMT, we also examined the effects of the procedure on the abundance and dynamics of individual bacterial taxa within the four original CDI patients. As shown previously [2-8], the relative abundance of bacterial phyla in patient fecal samples shifted substantially following FMT, with relative decreases in *Proteobacteria* and relative increases in *Bacteroidetes* and *Firmicutes* (Figure 3). These *Proteobacteria* are primarily the order *Enterobacteriales*, which were also substantially decreased in relative abundance following FMT (Figure 4A).

We focused on these changes by examining the relative abundance of *Enterobacteriales* alone in each patient before and after FMT. The relative abundance of this taxon ranged from 44% to 82% in all four patient samples prior to FMT and rapidly dropped to undetectable levels within 1 week after the procedure. Moreover, abundance of this taxon remained low at 26 days after FMT, the latest time point shared by all four patients (Figure 4A), although other members of the *Proteobacteria* remain detectable if decreased in relative abundance (Figure 3). In addition, we generated individual value control charts based on the average abundance of this taxon in recurrent CDI patients. Compared to relative abundance, these control charts displayed the expected variation of the abundance of *Enterobacteriales* in these fecal samples. In all patients, the abundance of *Enterobacteriales* was above the expected variation (that is, more than three standard deviations above the mean relative abundance [the standard upper control limit, or UCL] of this order across all samples) prior to FMT, and rapidly fell below the upper control limit within 1 to 2 days after the procedure (Figure 4B). These results suggest that the relative abundance of *Enterobacteriales* significantly decreased in all patients soon after FMT to levels similar to donor samples and remained within a statistically expected range for the duration of sample collection (up to 151 days post-FMT).

### Post-FMT communities are initially similar to donor samples but can later diverge

**Figure 5:**
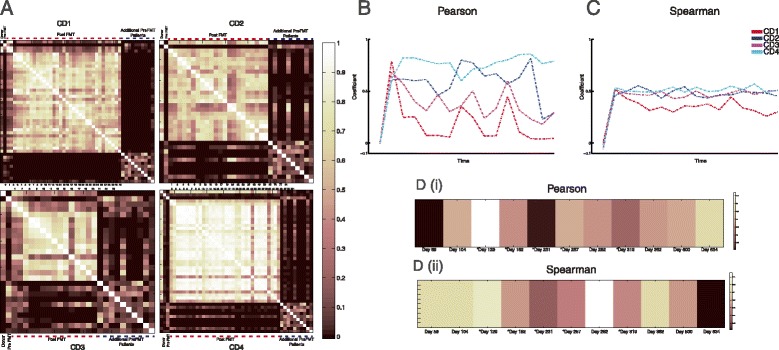
**Pearson and Spearman correlations between fecal communities before and after FMT. (A)** Heat map of Pearson correlation values between each sample within each patient set, corresponding donor, and 10 additional pre-FMT patient samples (far right). **(B)** Pearson correlation values between donor sample and each patient sample. **(C)** Spearman correlations between donor sample and each patient sample. **(D)** Heat maps of Pearson (i) and Spearman (ii) correlation values between earliest donor sample and eleven subsequent samples; days represent collection time of each sample versus earliest donor sample. CD1 to CD4, patients 1 to 4. Dashed lines indicate samples after FMT.

Next, we compared fecal microbial communities within each patient over time to that of the initial donor sample. We generated heat maps based on Pearson correlations between every sample within a given patient set, including respective donor samples and samples from 10 additional pre-FMT patients (Figure 5A). This analysis revealed that while microbiota in samples from patients after FMT quickly became similar to microbiota in donor samples, the similarity of samples taken at later time points after FMT fluctuated.

To further investigate how fecal microbial communities in these patients correlate to donor communities, we examined Pearson and Spearman correlations between donor and patient samples, which were common to each patient (pre-FMT samples and those up to 26 days post-FMT; Figure 5B,C and Additional file 4: Figure S2). While fecal microbial communities from patients before FMT were highly distinct from those in the donor, fecal microbial communities from samples 1 day after the procedure were highly correlated to donor communities *via* both Pearson and Spearman analyses in all patients. After the initial time point after FMT, the Pearson correlation values of patient to donor samples were highly variable within and across patients, although Spearman correlations remained high for three patients. To examine whether this variation is similar in healthy individuals, we determined Pearson and Spearman correlations within the four donor samples used in FMT, as well as eight additional donor samples from the same individual as a control. Results of this analysis revealed that donor microbiota also changed over time (Figure 5D). These findings suggested that the level of variability seen across patient post-FMT fecal microbial communities was within the range of normal microbiota behavior in a healthy individual.

### Normalization and dynamic range of post-FMT patient fecal microbial communities are similar to donor communities

Because of the observed variability in later post-FMT patient fecal communities relative to single donor communities, we compared the communities of these patient samples to an expanded set of 17 samples taken from the same donor. We generated two metrics to evaluate the relationships between these communities: normalization and dynamic range (stability). Normalization refers to the mean between-sample distance for each set of patient samples versus the set of donor samples, while dynamic range is the mean distance between each sample within a single patient set. Effectively, the normality of a post-FMT patient sample set is a measure of how similar it is to the donor (healthy) sample set, while dynamic range is a measure of variability within a given patient sample set. We found that neither the normalization nor the dynamic range of any post-FMT patient sample set was significantly different than the donor set following analysis using unweighted UniFrac (Table 1). This suggested that although fecal microbial communities of patients post-FMT do not remain identical to the donor, they nonetheless fall within expected parameters relative to the healthy donor. Similar results were obtained when these analyses were repeated with other parameters, including weighted UniFrac, Jensen-Shannon and root Jensen-Shannon, and Bray-Curtis (data not shown).

**Table 1:** ***P*** **values of normalization and dynamic range of patient samples sets versus donor set**

**Patient**	**Normalization**	**Dynamic range**
CD1	0.154	0.484
CD2	0.429	0.429
CD3	0.165	0.308
CD4	0.484	0.473

## Discussion

It is now well understood that the fecal microbiota change substantially following FMT, typically shifting to fecal microbial communities more similar to those of the donor after transplant [2-8]. Here we show that these communities shift away from a dysbiotic state towards a composition that is representative of fecal microbial communities from hundreds of healthy individuals, collected in the HMP [19]. Similarly to previous studies [4-8], the dysbiotic state in these patients with multiply recurrent CDI is characterized by a large expansion of *Proteobacteria* (primarily members of the order *Enterobacteriales*, which contains the family *Enterobacteriaceae*), and FMT is associated with reemergence of dominance by members of the *Bacteroidetes* and *Firmicutes* phyla.

Analysis of multiple donor and post-FMT samples demonstrates the dynamic behavior of fecal microbial communities over time. Both donor and recipient samples are characterized by highly dynamic shifts that nonetheless remain within the compositional range of normal fecal microbiota. This observation is consistent with known rapid responsiveness of the fecal microbiome to environmental inputs, such as dietary variations [11], and drifts in microbiota composition over time in healthy individuals [22].

The dynamic nature of intestinal microbiota is an intrinsic property, which should be taken into account when considering how therapeutic interventions, including FMT, impact its composition over time. In long-term post-FMT follow-up, Song and colleagues also noted dynamic changes in the fecal microbiome of R-CDI patients up to 16 weeks post-FMT [9]. These investigators concluded that the fecal microbiome of post-FMT patients did not fully recover over this time, despite clinical recovery. Indeed, we observed divergence of microbiome in some of the patients away from the original implanted material over time. However, analysis of multiple donor samples showed that this movement is within the same dynamic range observed in the donor’s fecal microbiome. We therefore conclude that the dynamic behavior of microbiota needs to be taken into account in making comparisons between individuals, and should become an integral part of analysis of the success of FMT.

Three of the recipients in this study received freshly prepared microbiota, while one received frozen/thawed preparation. Use of frozen microbiota preparations is increasing in clinical practice [23], and its equivalency has not been rigorously established in randomized clinical trials. The ability to store microbiota allows the most up-to-date testing of the donor and fecal material for infectious pathogens, as some of the current tests may take several weeks to complete. Therefore, ability to preserve donor microbiota long-term is critical for its development as a therapeutic agent in clinical practice. Our results here, although limited in the number of patients, demonstrate indistinguishable behavior of fresh and frozen/thawed microbiota preparation.

The patients in this study did not have any significant gastrointestinal comorbidities. However, a significant proportion of patients with recurrent CDI have underlying inflammatory bowel disease, take potent immunosuppressive medications, or have multiple other medical problems [18,24]. The importance of these host factors in contributing to microbiota behavior is currently unknown, but is a subject of great interest [25]. Understanding these influences will require analysis of multiple samples. Recently, Fuentes and colleagues [8] reported that some specific microbial groups and interactive networks are likely to be very important for the maintenance of microbiota in healthy individuals. However, although there is a great deal of effort focused on discovery of compositional differences in microbiota between normal subjects and individuals with different gastrointestinal and medical conditions, the dynamic behavior of fecal microbiota constitutes another dimension that may distinguish these cases. Thus, predictors of stable or dysbiotic intestinal microflora may also change over time. Further detailed studies of dynamic behavior of post-FMT microbiota may improve our understanding of causal connections between microbial communities and different disease states.

## Conclusions

The fecal microbiota of patients with R-CDI continues to undergo change after FMT is performed, though these changes appear to fall within the range of normal variation of healthy individuals over time. Dynamic behavior is an intrinsic property of normal fecal microbiota and should be accounted for in comparing microbial communities among normal individuals and those with disease states.

## Methods

### Patients and donors

All patients suffered from multiply recurrent CDI refractory to standard antibiotic therapies. A single standard donor was used in the preparation of all fecal microbiota material as described previously [18]. The Institutional Review Board at the University of Minnesota approved prospective collection of fecal specimens and their analysis. All patients satisfied the inclusion criteria for the FMT within our program, which included at least two spontaneous recurrences of CDI within a month of discontinuation of antibiotics and failure of at least one advanced antibiotic regimen such as a vancomycin pulse/taper protocol or vancomycin treatment followed by administration of rifaximin or fidaxomicin for 2 to 3 weeks. The specific clinical characteristics of patients involved in this study are summarized in Additional file 5: Table S2.

### Fecal microbiota transplantation

FMT was done using a standardized preparation of concentrated fresh or frozen fecal bacteria *via* colonoscopy as previously described [18]. All patients were treated with oral vancomycin, 125 mg four times daily, until 2 days prior to the procedure [18]. The day before the procedure, patients received a polyethylene glycol-based colonoscopy prep (GoLYTELY® or MoviPrep®) to remove residual antibiotics and fecal material. Donor fecal microbiota was placed into the terminal ileum and/or cecum *via* the biopsy channel of the colonoscope. A total of 17 donor samples from the same individual were used in these studies. The CD1 to CD4 donor samples were given to patients CD1 to CD4, respectively. Patients CD1, CD3, and CD4 received freshly prepared fecal microbiota, while patient CD2 received a previously frozen preparation of fecal microbiota, all from the same standardized, anonymous donor.

### Sample collection

Fecal samples were collected at home by the patients using swabs to sample feces deposited into a toilet hat immediately after production and stored frozen at approximately −20°C. Samples were subsequently transferred to the laboratory and stored at −80°C until used. Donor samples for DNA extraction were collected during processing of material for FMT and stored frozen at −80°C until used. Samples from patients CD1 to CD4 were obtained prior to FMT and between 1 to 151 days post-FMT, with daily collection until day 28, and weekly collection until day 84. Fecal material prior to FMT was obtained from patients CD5 to CD14.

### DNA extraction

DNA was extracted from donor and recipients’ pre- and post-FMT fecal samples using MOBIO PowerSoil DNA extraction kits (MOBIO, Carlsbad, CA, USA), according to the manufacturer’s instructions. Fecal DNA concentrations were measured using a QuBit DNA quantification system (Invitrogen, Carlsbad, CA, USA).

### PCR amplification

Extracted DNA was amplified using the EMP standard protocols at http://www.earthmicrobiome.org/ following the recommendations of Caporaso *et al*. [26]. Briefly, F515/R806 primers were used, with 12-base Golay codes introduced on the 806 end to provide unique sample indices. Cycling and annealing conditions were as previously described [26].

### DNA sequencing

DNA sequencing was performed as previously described [26] on an Illumina MiSeq platform using 2 × 150 bp paired-end reads and the Illumina v3 reagent chemistry.

### Sequence processing and analysis

Sequence data was processed and analyzed using QIIME [21] according to the Illumina demultiplexing and processing protocol [26] and current quality-filtering recommendations [27], using the 1.8.0 pipeline and the default parameters in split_libraries_fastq.py. After quality control and demultiplexing, we picked close references at 97% similarity against the 97% similarity Greengenes database [28] version 13_8. All further analyses were performed at a rarefied depth of 5,000 reads/sample. EMPeror [29] was used for data visualization of BIOM-format [30] OTU tables. OTU analyses were performed by clustering at the 97% level with UCLUST [31], and data were integrated with the HMP dataset according to the protocols used for similar previous meta-analyses [15,32]. Sequences were analyzed by using both weighted and unweighted UniFrac [32], followed by principal coordinate analysis [21]. Data were visualized using Phinch. The Phinch program provides an easy-to-use, browser-based, platform to visualize contingency tables along with their sample metadata (Bik *et al*., manuscript in preparation, https://github.com/PitchInteractiveInc/Phinch).

### Analysis of microbiome stability and centrality

For each set of post-transplant patient samples, we assessed the similarity of that set to the set of reference samples from the donor (2,000 reads/sample). To reduce noise and compare patient samples along only relevant dimensions in UniFrac distance space, we applied PCoA to the unweighted UniFrac distance matrix containing only the post-transplant and donor samples for that donor-patient pair, then recalculated the distances using only the first *n* principal coordinates axes required to explain at least 80% of the variation in the distance matrix. An 80% cutoff was chosen to balance bias and overfitting. Distances were recalculated using Euclidean distances between points in PCoA space in order to convert PCoA coordinates to a distance matrix. The empirical *P* values for the ‘normality’ were obtained by comparing the mean distance between patient and donor samples to the histogram of within-donor distances (generated using all samples from a given donor by enumerating the pairwise distance between those samples). The empirical *P* values for the ‘dynamic range’ (stability) were obtained by comparing the mean distance within patient samples to the histogram of within-donor distances. These analyses were also performed using alternative parameters including, weighted UniFrac, Jensen-Shannon, root Jensen-Shannon, and Bray-Curtis.

## Consent

Approval for this study was given by the University of Minnesota Institutional Review Board (Protocol Number: 0901M56962). All human subjects provided informed consent for participation in the study and collection and analysis of data. All human subjects gave their permission for their information to be published.

## Additional files

Additional file 1: Movie supplement. An animated visualization of FMT-associated changes in patients’ fecal microbial communities. Additional file 2: Figure S1. Microbial communities remain dynamic after FMT. (A) Unweighted and (B) weighted UniFrac analyses, followed by principal component analysis of bacterial communities of recurrent CDI patient fecal samples, by time point after FMT and donor samples (blue). PC: principal component. Percentages represent percent variability explained by each principal component. See key at right for colors associated with samples from patients after FMT (CD1-CD4). Additional file 3: Table S1. OTU table. Additional file 4: Figure S2. Pearson and Spearman correlations between fecal communities before and after FMT for all collected fecal samples. Heat maps indicating Pearson (left) and Spearman (right) correlation values between respective donor and pre- or post-FMT fecal microbial communities of patients. Additional file 5: Table S2. Clinical metadata of patients used in this study.

## Abbreviations

CDI: *Clostridium difficile* infection
FMT: fecal microbiota transplantation
HMP: Human Microbiome Project
LCL: lower control limit
OTU: operational taxonomic unit
PCoA: principal coordinates analysis
R-CDI: recurrent *Clostridium difficile* infection
UCL: upper control limit
