# Supplementary figures and images for: Dynamic changes in short- and long-term bacterial composition following fecal microbiota transplantation for recurrent Clostridium difficile infection
Source: Microbiome. 2015 Mar 30;3:10. doi: 10.1186/s40168-015-0070-0 (PMC4378022; doi:10.1186/s40168-015-0070-0)

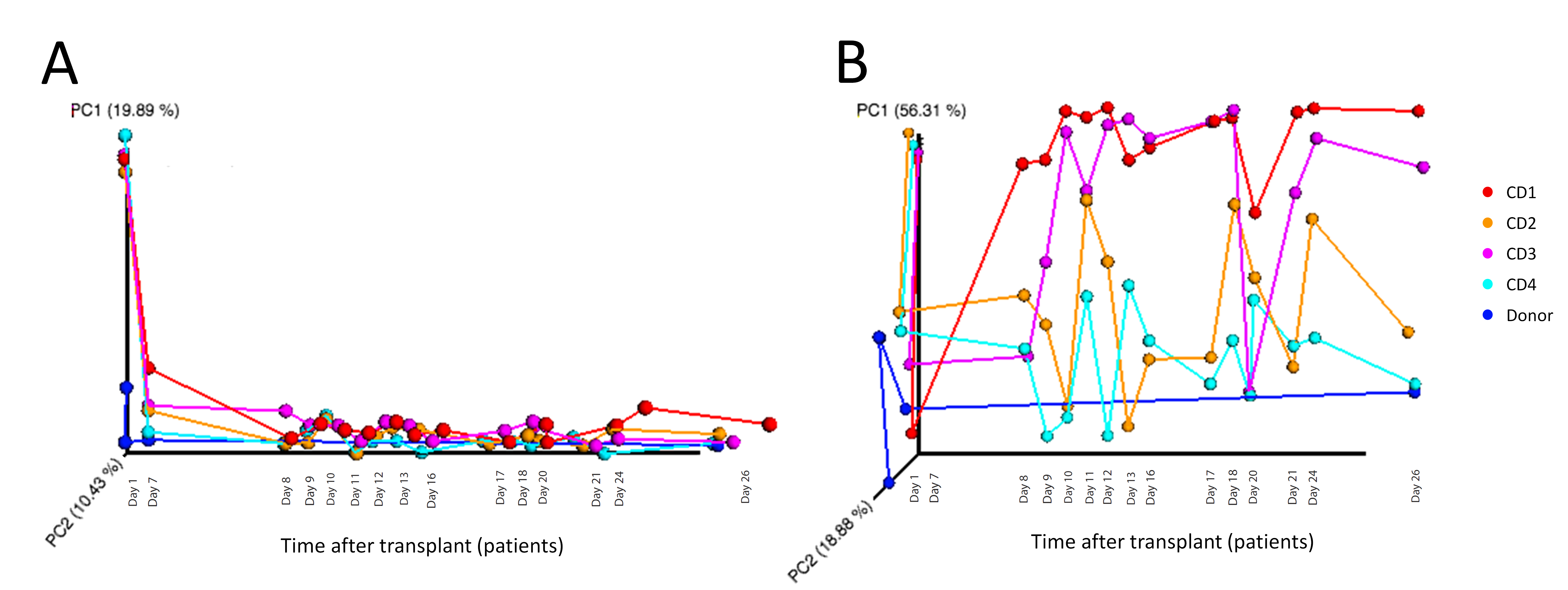

Supplement: Additional file 2: Figure S1. — Microbial communities remain dynamic after FMT. (A) Unweighted and (B) weighted UniFrac analyses, followed by principal component analysis of bacterial communities of recurrent CDI patient fecal samples, by time point after FMT and donor samples (blue). PC: principal component. Percentages represent percent variability explained by each principal component. See key at right for colors associated with samples from patients after FMT (CD1-CD4). [file 40168_2015_70_MOESM2_ESM.png]

# Pearson

# Spearman

CD1

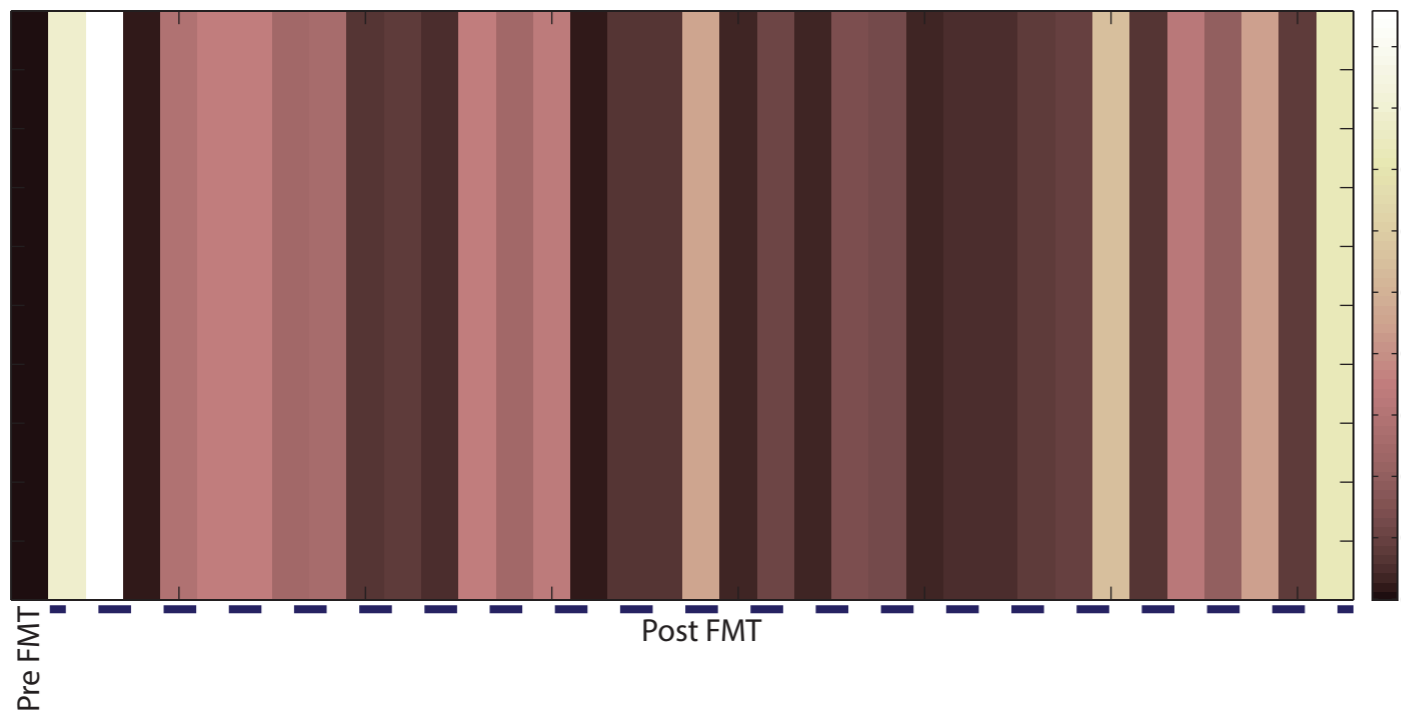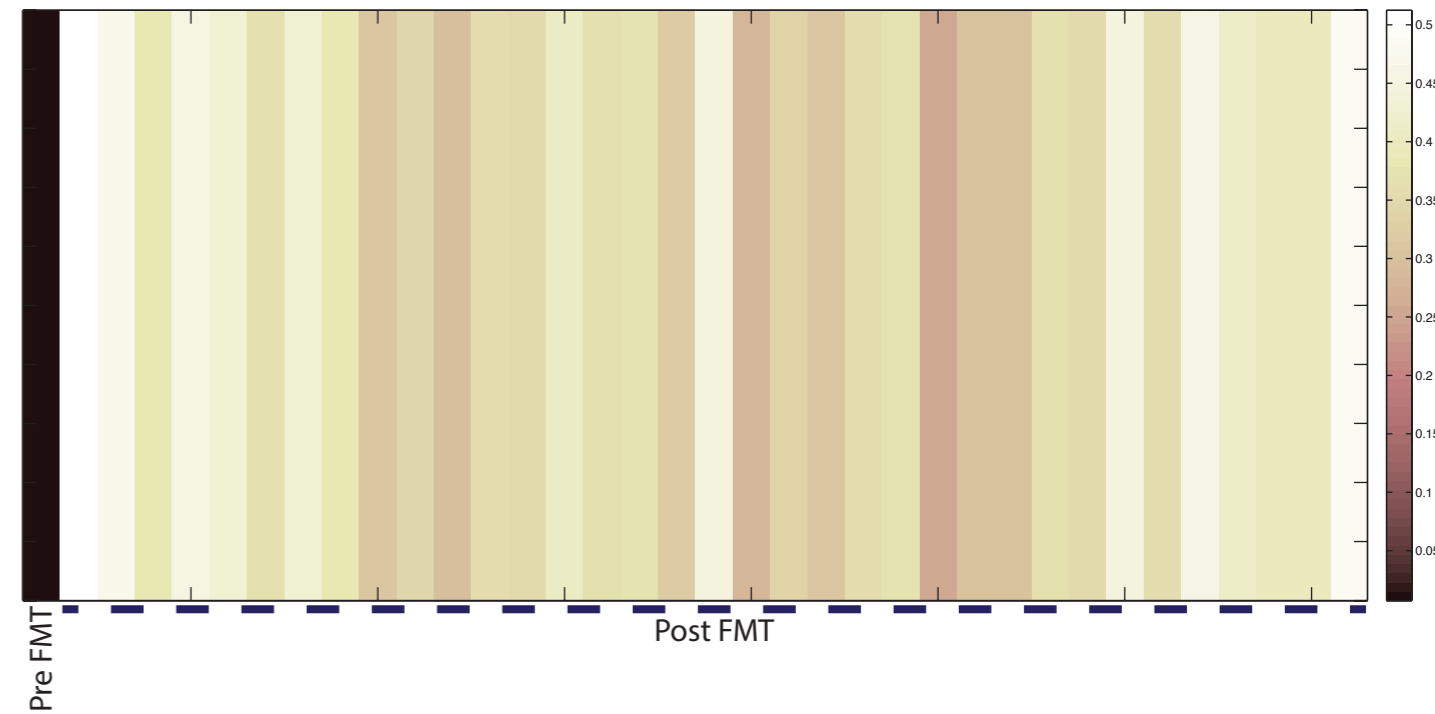

CD2

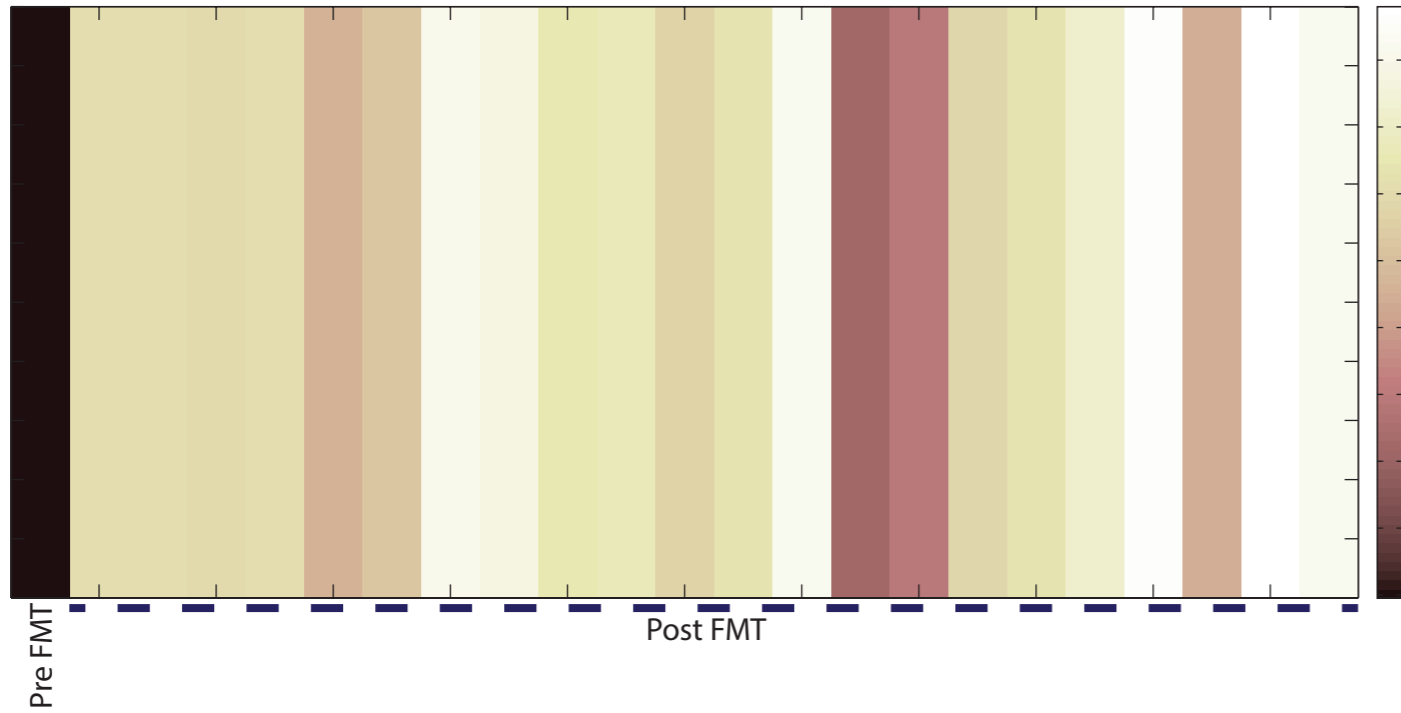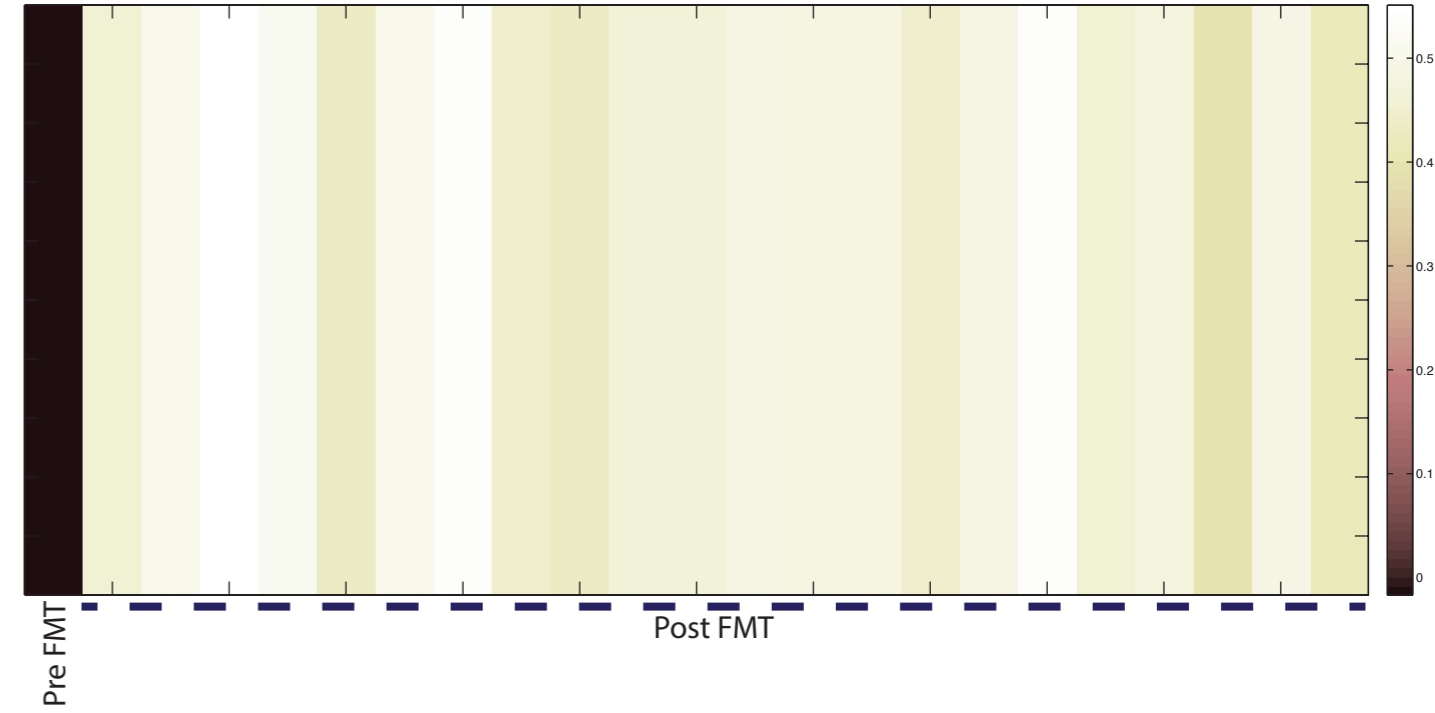

CD3

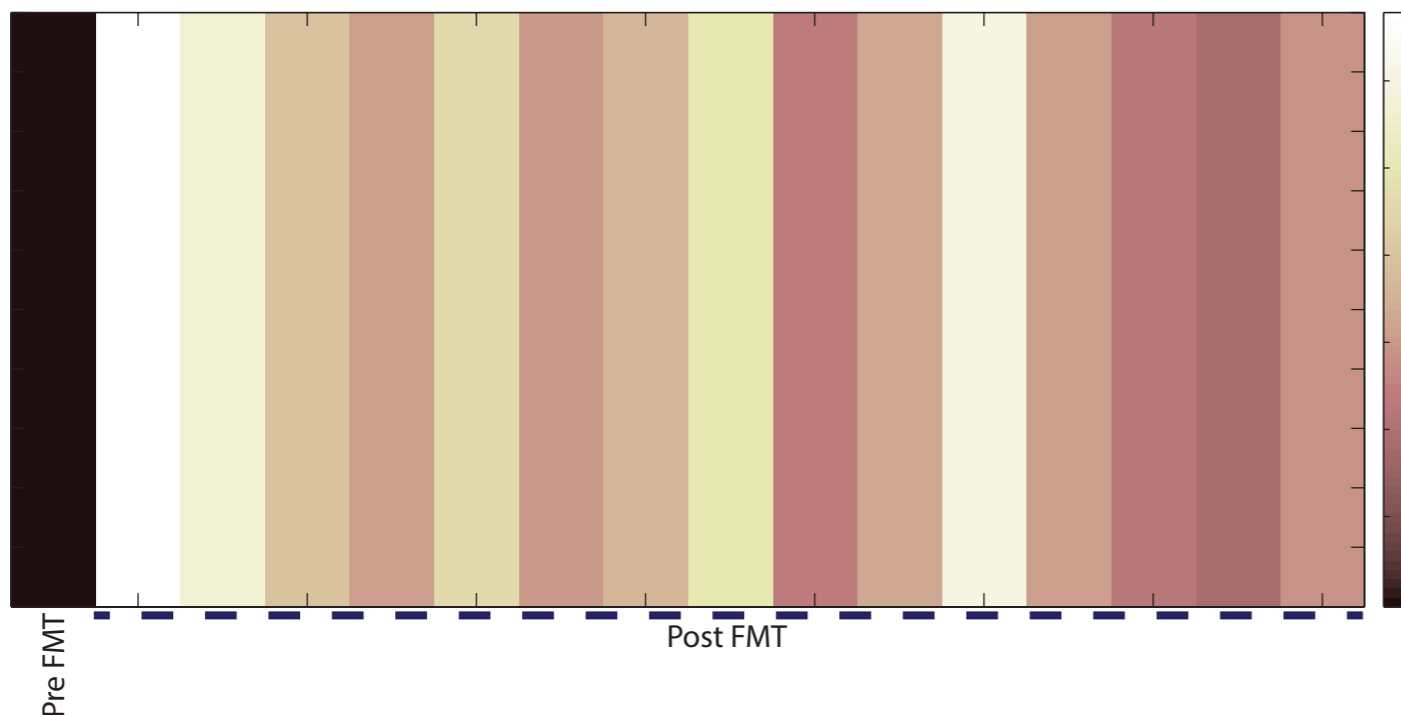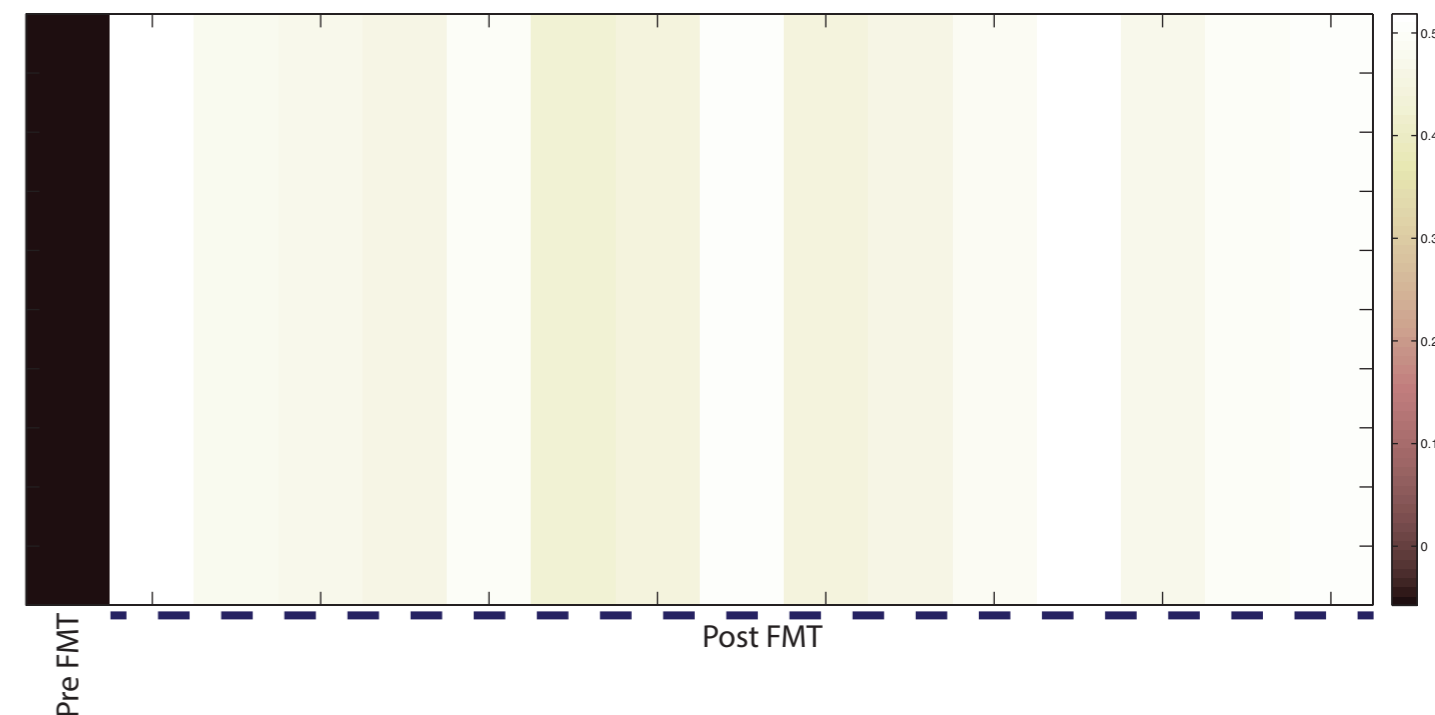

CD4

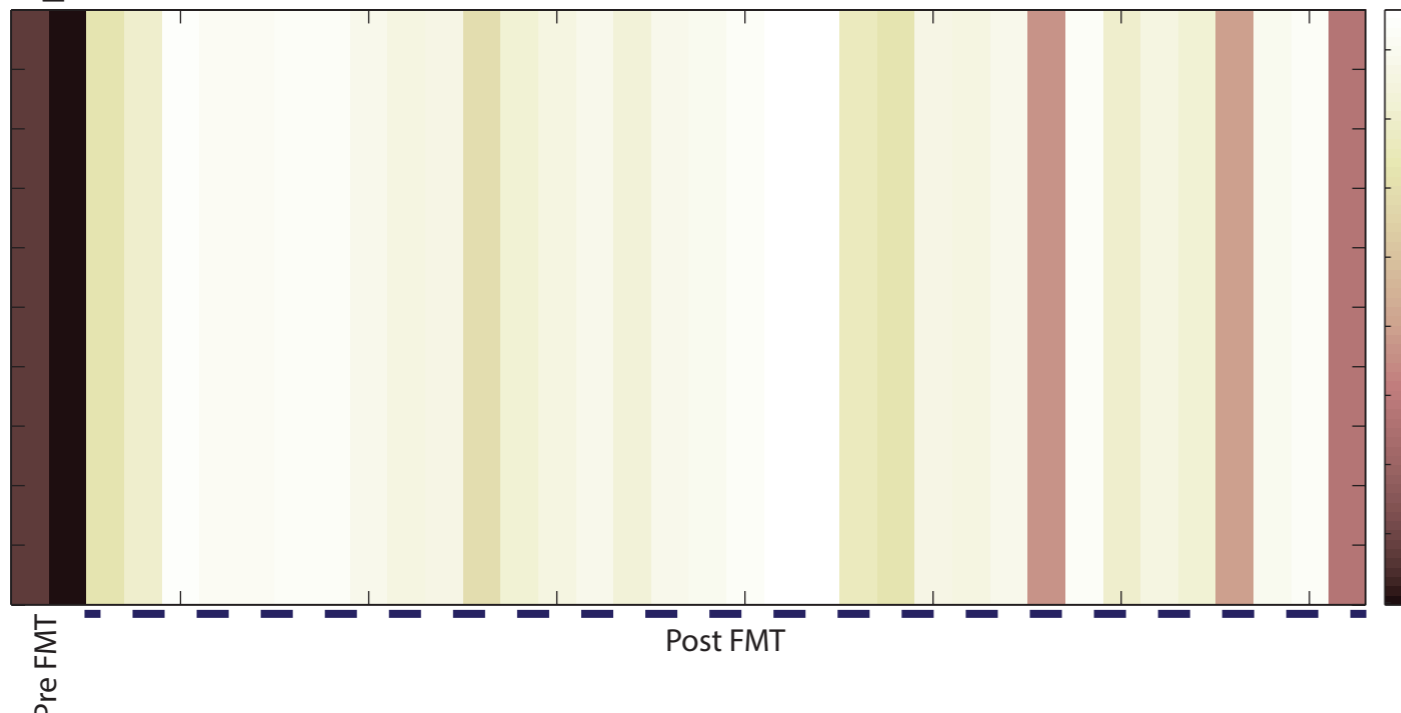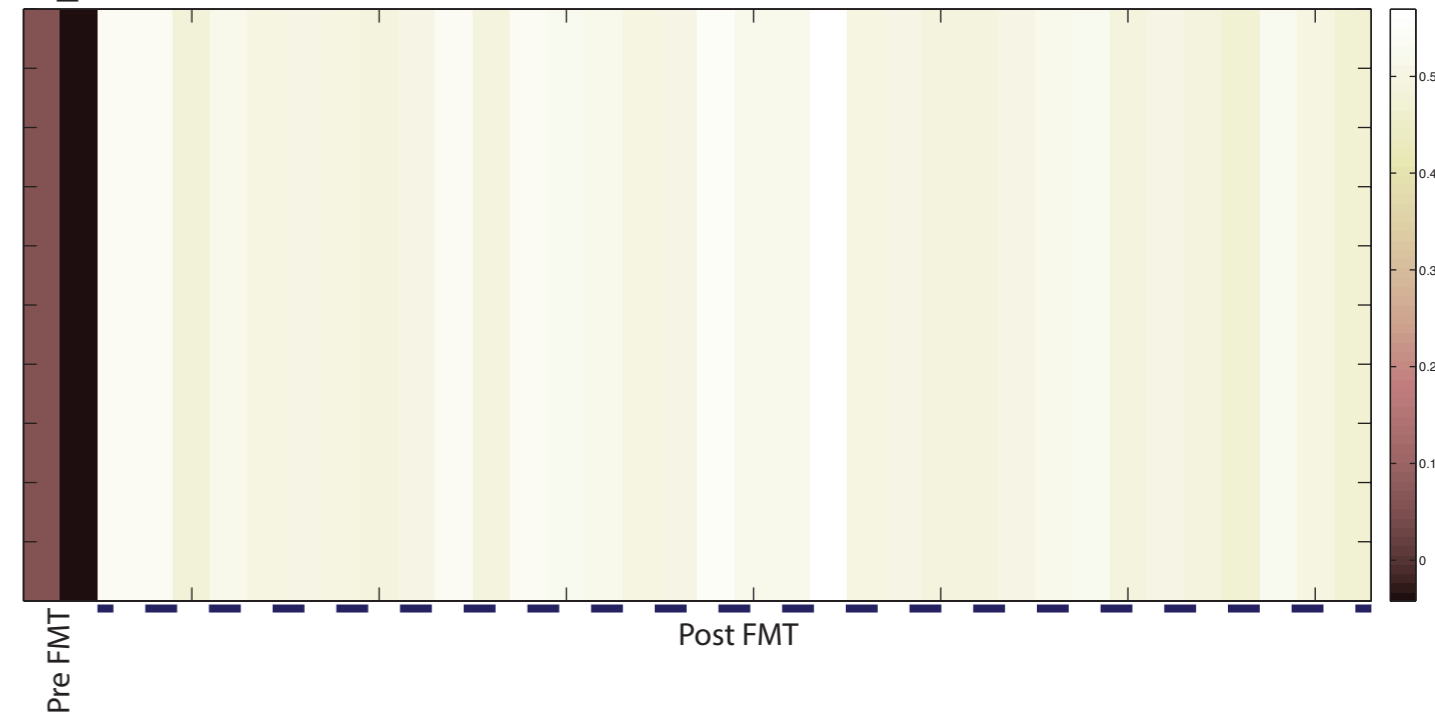

Supplement: Additional file 4: Figure S2. — Pearson and Spearman correlations between fecal communities before and after FMT for all collected fecal samples. Heat maps indicating Pearson (left) and Spearman (right) correlation values between respective donor and pre- or post-FMT fecal microbial communities of patients. [file 40168_2015_70_MOESM4_ESM.pdf]
