# Supplementary material for: Dynamic changes in short- and long-term bacterial composition following fecal microbiota transplantation for recurrent Clostridium difficile infection
Source: Microbiome. 2015 Mar 30;3:10. doi: 10.1186/s40168-015-0070-0 (PMC4378022; doi:10.1186/s40168-015-0070-0)
Supplement: Additional file 5: Table S2. — Clinical metadata of patients used in this study. [file 40168_2015_70_MOESM5_ESM.docx]

**Additional file 5: Table S2. Clinical metadata of patients used in this study.**

| Patient | Age | Gender | Trigger Event | Trigger Antibiotic | Duration of RCDI prior to FMT (months) | Co-morbidities; History of GI Surgery | History of Treatment of CDI with Metronidazole | History of Treatment of CDI with Vancomycin | History of Treatment of CDI with Rifaximin | History of Treatment of CDI with Fidaxomicin | History of Trreatment with Nitazoxanide |
| --- | --- | --- | --- | --- | --- | --- | --- | --- | --- | --- | --- |
| CD1 | 39 | F | Antibiotics with Caesarian section | N/A | 18 | None | Yes | Yes | Yes | No | Yes |
| CD2 | 52 | M | Sinusitis | N/A | 5 | None | Yes | Yes | Yes | No | No |
| CD3 | 55 | M | Antibiotics with a dental procedure | N/A | 18 | Immunoglobulin deficiency secondary to administration of rituximab; s/p cholecystectomy | Yes | Yes | Yes | No | No |
| CD4 | 60 | F | Diverticular abcess | Ciprofloxacin and Metronidazole | 7 | None | Yes | Yes | No | No | No |
| CD5 | 83 | F | Sepsis (*Proteus mirabilis*) | N/A | 8 | None | Yes | Yes | No | No | No |
| CD6 | 71 | M | N/A | N/A | 7 | Diverticulosis | Yes | Yes | No | No | No |
| CD7 | 66 | F | Antibiotics with spinal surgery | N/A | 3 | S/P Billroth II gastrectomy; Diverticulosis | Yes | Yes | No | No | No |
| CD8 | 72 | M | Pneumonia | Trimethoprim/sulfamethoxazole; vancomycin; levofloxacin; meropenem | 13 | S/P heart transplant; Liver cirrhosis due to hepatitis B; Diverticulosis | Yes | Yes | No | No | No |
| CD9 | 65 | F | Vaginal infection | Clindamycin | 7 | S/P appendectomy | Yes | Yes | Yes | No | No |
| CD10 | 47 | F | Upper respiratory tract infection | Clindamycin | 3 | S/P cholecystectomy; Lymphocytic colitis | Yes | Yes | Yes | No | No |
| CD11 | 36 | M | N/A | N/A | 8 | None | Yes | Yes | No | No | No |
| CD12 | 52 | F | Antibiotics with hysterectomy | N/A | 5 |  | Yes | Yes | Yes | Yes | No |
| CD13 | 56 | F | Antibiotics with knee replacement | N/A | 3 | None | Yes | Yes | No | Yes | No |
| CD14 | 53 | F | Urinary tract infection | Ciprofloxacin | 8 | None | Yes | Yes | No | Yes | No |
